# Data on the diet and nutrition of urban and rural bumblebees

**DOI:** 10.1038/s41597-025-04585-w

**Published:** 2025-02-17

**Authors:** Joan Casanelles-Abella, Simonetta Selva, Alexander Keller, Fabian A. Ruedenauer, Bertrand Fournier, Sara D. Leonhardt, Marco Moretti

**Affiliations:** 1 Urban Productive Ecosystems, TUM School of Life Sciences, Technical University of Munich, 85354 Freising, Germany; 2 Biodiversity and Conservation Biology, Swiss Federal Research Institute WSL, 8903 Birmensdorf, Switzerland; 3 Plant Ecology, Department of Environmental Sciences, ETH Zurich, 8092 Zurich, Switzerland; 4 Cellular and Organismic Networks, Faculty of Biology, Ludwig-Maximilians University Munich, 82152 Munich, Germany; 5 Plant-Insect Interactions, TUM School of Life Sciences, Technical University of Munich, 85354 Freising, Germany; 6 Landscape Ecology, Institute of Environmental Sciences and Geography, University of Potsdam, 14476 Potsdam, Germany

**Keywords:** Behavioural ecology, Animal behaviour, Urban ecology, Agroecology

## Abstract

Land-use changes, driven by agricultural intensification and urbanization, are major contributors to biodiversity loss, altering habitats and reducing available resources. These changes impact species’ foraging strategies, particularly in human-modified ecosystems. While dietary shifts due to land-use changes have been well-studied in vertebrates, similar research in invertebrates, such as wild bees, remains limited. The present data paper provides a comprehensive dataset on the pollen collected from urban and rural populations of two bumblebee species (*Bombus lapidarius* and *B. pascuorum*) in Switzerland, examining pollen composition, nutrient content, and diet breadth. Additionally, by analyzing pollen from both body and leg-baskets, the dataset also offers a comprehensive overview of plant-bumblebee interactions. The data help understand plant-bumblebee interactions, pollination services, nutritional supply to larvae, and the impact of land-use changes on these processes. Furthermore, the dataset can be integrated with existing plant trait data to explore the effects of non-native species and other ecological factors on bumblebee foraging and nutrition in anthropogenically modified landscapes.

## Background & Summary

Land-use changes, in the form of agricultural and urban conversion, and the resulting anthropogenic ecosystems have consequences on eco-evolutionary processes and biodiversity. Increases in land-use intensity, such as agricultural expansion and intensification, and urban sprawl and intensification, modify the available habitat and existing resources, by reducing or impoverishing them but also, by generating novel forms of habitat and foraging landscapes^1–3^. Therefore, the altered ecological conditions of anthropogenic ecosystems shape eco-evolutionary patterns as well as biodiversity across organisational levels, resulting, for example, in new genotypes and phenotypes^4–6^.

The ecological properties of anthropogenic ecosystems are not uniform and can vary substantially. Urban and rural areas, the main forms of human-modified ecosystems, diverge in many of their ecological properties^7–9^. On the one hand, rural areas typically have a lower proportion of impervious surfaces, and therefore, have larger vegetated covers. However, habitat heterogeneity can be substantially low in highly intensified agricultural systems^10,11^, dominated by few cultivated plants and with reduced amounts of other habitat types^11,12^. On the other hand, urban areas can have more fragmented patches and resources due to increasing coverage of impervious surfaces and buildings, particularly in highly urban intensified parts of a city^13,14^. However, habitat heterogeneity is often large in cities^15,16^, and also, urban habitats are often dissimilar in terms of their plant assemblages due to different degrees of human-investment^17^. Moreover, urban habitats have larger representations of ornamental and non-native species^18,19^. Overall, distinct plant communities are expected to impose different challenges for taxa that depend on them such as pollinators, potentially modifying their interaction networks, their feeding behaviour and, by extension, their dietary patterns^20–25^. Dietary patterns include species’ diet breadth, which is the range or diversity of food items that an organism consumes, and species’ nutrient intake, which is for instance, the content or ratios of key macronutrients such as carbohydrates, proteins and lipids^26,27^. While these two aspects of the dietary patterns can be positively related, for instance, as predicted by the diversification hypothesis^28,29^, where a larger diet breadth translates into a better nutrition, this is not a given^30^. The combination of both diet breadth and nutrient intake provides a complementary interpretation on the foraging strategies of taxa and how they might be modified according to different environmental conditions^31,32^. Finally, changes in the dietary patterns have been studied in several vertebrate species (e.g.^20,33,34^,), yet they remain more pervasive in invertebrates.

Bumblebees are a good study system to monitor the influence of land-use changes in the foraging strategies and dietary patterns. Bumblebees are central-place foragers, foraging within the vicinity of their colony, and hence, their dietary patterns can be good indicators of the surrounding environmental conditions, potentially indicating signals of biotic or abiotic stress and disturbance^35–40^. Moreover, as vectors of pollinators, the dietary patterns of bumblebees can also be used to understand plant-bumblebee interactions and thus, pollination functions and associated effects on plants^41^ and contributions to people. While the dietary patterns have been investigated in some bumblebee species, including the widespread Bombus terrestris in Europe^42,43^ and Bombus impatiens in the USA^21,44^, as well as other generalists and specialist species in different ecosystems^22,38,40^, there still remains major lack of datasets describing the diet of multiple species in different ecosystems, particularly anthropogenically-modified ones (e.g., cities). Documenting the diet in anthropogenically-modified ecosystems is thus critical to understand how species perform under novel ecological conditions as well as for informing preservation actions^31,45^.

Bumblebees transport pollen via two structures, their body and their leg-baskets, with different results. One the one hand, pollen collected in the leg-baskets is mostly used for feeding the larvae, and thus, is often expected to be carefully selected (i.e., be more conserved) to reach the potential nutritional requirements and constraints that a species might have^42,44,46,47^. On the other hand, body pollen can have a more heterogenous origin: not only from targeted plants for larval pollen, but also coming from plants targeted for nectar feeding (as all workers need to fuel their flights^48^), as well as from the contact with non-targeted plants. In this last case, that can be the result, for example of random contacts between the bumblebee and the pollen (e.g., air, other pollinating animals carrying pollen) or due to foraging learning processes, with inexperienced bumblebees exploring what are suitable floral hosts^49,50^. Despite the complementary information provided from the two pollen transportation structures, most studies only focus on one of them.

Here, we provide a comprehensive dataset on the pollen collected in two transportation structures (i.e., the body and the leg-baskets) of the urban and rural populations of two bumblebee species (i.e., *Bombus lapidarius* and *Bombus pascuorum*) in Switzerland. Our dataset emerges from a combination of pollen metabarcoding and nutritional chemical analyses. Particularly, our dataset contains information on (1) the plant composition found in the body and leg-baskets with their relative abundances, (2) the content and ratios of two macronutrients, that is, amino acids and fatty acids, and (3) taxonomic, functional and phylogenetic metrics depicting the larvae’s diet breadth from the pollen collected in the leg-baskets. Overall, our data depicts critical information on the dietary patterns and pollen transportation patterns of urban and rural bumblebees.

Our data can facilitate understanding of different biological and ecological questions regarding bumblebees in human-modified ecosystems. First, our data provide information on the dietary patterns of two different species of bumblebees, which is relevant in itself for better knowing the biology and ecology of these species, and that can be used with other available datasets on the same species or different ones. Second, our data enable comparisons between urban and rural populations on a critical aspect of species’ ecology, that is, the dietary patterns including the diet breadth and the nutrient intake, particularly for the larval stages. Third, we sampled pollen from two different transportation structures, i.e., body and leg-baskets, providing complementary information on the plant-bumblebee interactions and diet, as well as on the pollen transportation services, and potentially pollination services, that bumblebees do. Fourth, as we have identified the vast majority of plant taxa to the species level, our data can be combined with existing datasets containing plant traits (e.g., nutrient contents^51,52^, floral accessibility^18^), enabling further exploration of the dietary patterns and pollen transportation in different human-modified ecosystems (e.g., measuring the prevalence of non-native species, of different growth forms, of different structural blossom classes^53^).

## Methods

### Study sites and bumblebee sampling

**Fig. 1:**
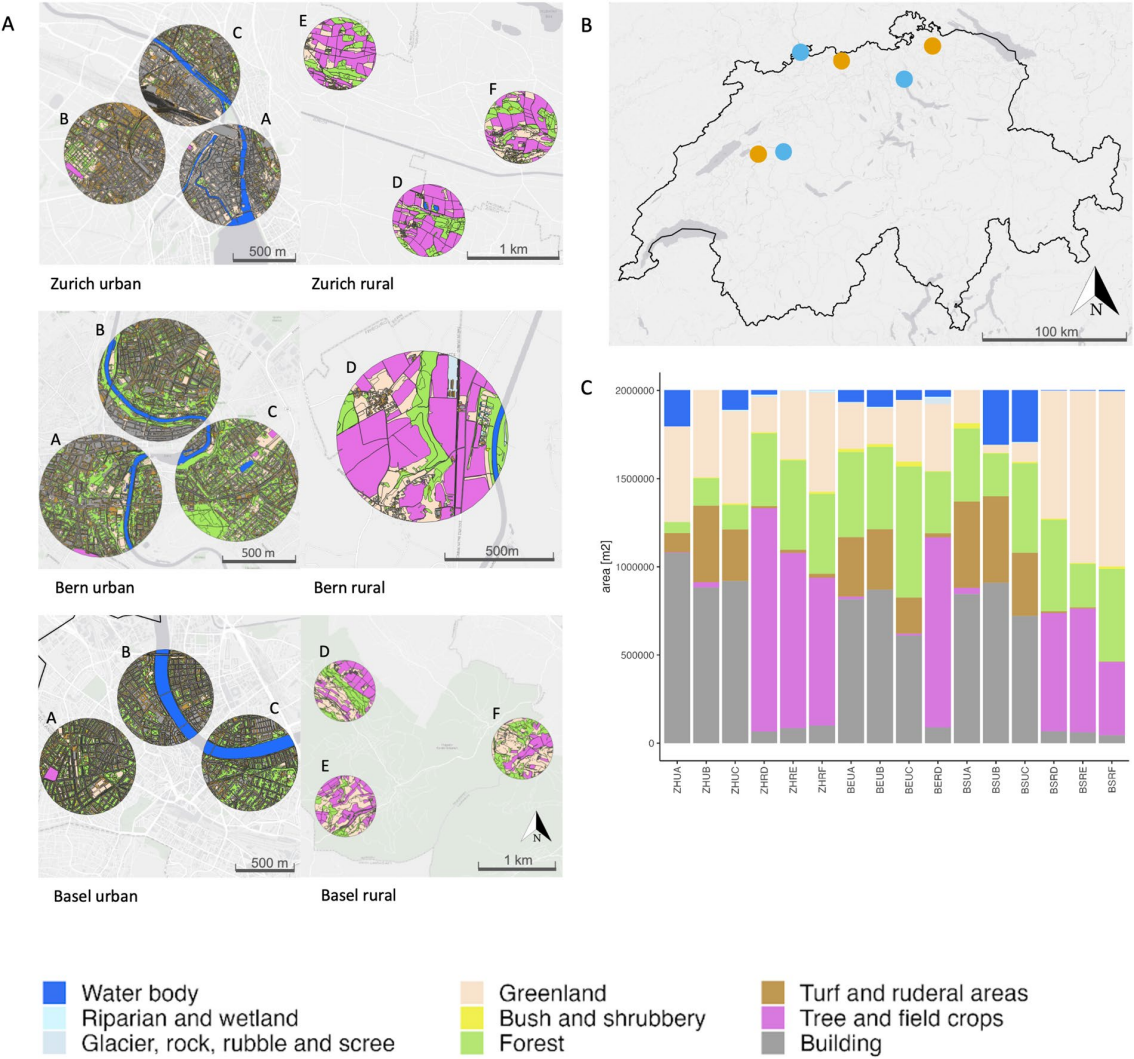
Sampling sites. (**A**) Configuration of the different habitats of the 16 sites across 6 regions sampled by Eggenberger *et al*.^54^ using the habitat map of Switzerland V1 (Price *et al*., 2021) in the cantons of Basel, Bern and Zurich. The 9 habitat tapes are indicated by colour. (**B**) Geographical distribution of the sampling regions in Switzerland (urban - blue, rural - orange). (**C**) Proportion of each habitat per site.

We conducted bumblebee sampling in both urban and rural areas across three Swiss regions (referred to as regions, Fig. 1), specifically within the Cantons of Basel, Bern, and Zurich^54^. For each region, three sampling sites were selected in urban areas (i.e., the cities of Basel, Bern, and Zurich) and in rural areas, except in rural Bern where only one site could be chosen (total = 16 sites, see section on Data records), following the methodology of Eggenberger *et al*.^54^. Specifically, urban sites were characterized by at least 60% impervious surfaces and were situated in the city centers, at least 1 km away from suburban areas and urban forests. Rural sites were standardized based on specific criteria: low-density settlements, meadow and pasture lands with similar management regimes, altitudes comparable to urban sites (400–600 m), proximity to water, and minimal forest cover. These rural sites were randomly selected within a defined area with a 4 km radius, representing the maximum extent of the urban centers. In each region, a minimum distance of 20 km separated urban and rural sites to minimize gene flow and interbreeding between bumblebee populations in these environments. The study encompassed 16 distinct sites: six around Zurich, six around Basel, and four around Bern. Within each region, we selected three non-overlapping sampling sites (radius of 800 m) that corresponded to the upper foraging ranges of the two target bumblebee species. Sampling began in the canton of Zurich, with the urban site in Zurich city (47°22′N, 8°33′E) and the rural site in the lower Töss valley (47°21′1′′N, 8°56′6′′E). The second region was in Basel, with the urban site in Basel-Stadt (47°34′N, 7°36′E) and the rural site in the Fricktal area in canton Aargau (47°30′21.43′′N, 8°3′7.63′′E). The third region was the canton of Bern, where the urban site was in the city of Bern (46°57′N, 7°27′E) and the rural site in the Bernese Mittelland (46°56′N, 7°12′E).

We focused on two bumblebee species, the common carder bee *Bombus pascuorum* (Scopoli, 1763) and the red-tailed bumblebee *Bombus lapidarius* (Linnaeus, 1761). Both species are widespread in the Swiss lowlands and occur in both urban and rural areas. While both are generalists, *B. pascuorum* has a longer tongue than *B. lapidarius*. Bumblebees were collected using hand-netting during the peak activity months for both species, from July to mid-August 2016. At each 800-m site, 30–40 individuals of each species were collected, with the exception of one urban site in Bern, where only three *B. lapidarius* individuals were found. Sampling efforts were standardized across all sites and conducted during peak bumblebee activity hours (09:00–17:00) under optimal weather conditions. We surveyed the entire 800-m radius for the target species but limited collection to a maximum of ten individuals per specific location within the site. Only active foragers were collected. Species identification of all collected individuals was confirmed in the laboratory, and specimens that could not be conclusively identified were excluded. Bumblebee samples were stored at -20 °C until pollen collection.

### Pollen collection

**Fig. 2:**
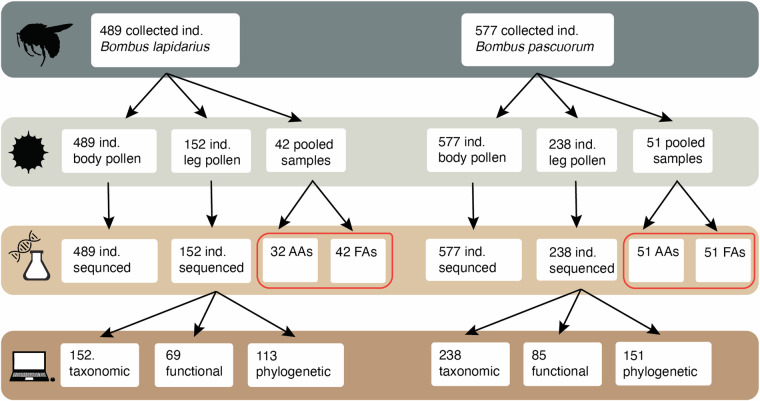
Summary of the workflow and gathered data for the two bumblebee species studied. The workflow is classified in four steps, from top to bottom: bumblebee individual collected, pollen extraction, metabarcoding and chemical analyses, and diet breadth calculations. For each step, the number of samples included is provided. From the starting number of individuals (489 individuals for *Bombus pascuorum* and 577 individuals in *Bombus lapidarius*) we used all individuals for the metabarcoding of pollen carried on the body of the bumblebees and a subset for the metabarcoding of the pollen carried in the leg-baskets (152 individuals in *B. lapidarius*, 238 individuals in *B. pascuorum*). Furthermore, we pooled the leg-basket pollen from different individuals from the same species collected in the same study site for the nutritional analyses (42 pooled samples for *B. lapidarius* and 51 pooled samples for *B. pascuorum*). From these samples, we uncovered the content and ratios of amino acids (AAs) and fatty acids (FAs), represented inside a red box in the figure. Finally, after obtaining the plant composition in the pollen collected in the leg-baskets, we estimated the diet breadth using taxonomic, functional and phylogenetic diversity metrics. Note that for B. lapidarius, we could only analyse the AAs in 32 out of 42 samples due to insufficient pollen mass.

We extracted pollen from the leg-baskets (i.e., corbicular pollen) and the body (i.e., body pollen) of the bumblebees (Fig. 2). For pollen collection, the bees were first removed from the -20 °C storage compartment.

#### Corbicular pollen

We extracted corbicular pollen in152 individuals of *B. pascuorum* and 238 individuals of *B. lapidarius* (Table 1, Fig. 2) across all sampling sites (Table 1, Fig. 2). To collect the corbicular pollen, the rear legs of each bumblebee were carefully detached under a binocular microscope, and the bee’s body was placed in a 2 ml Eppendorf tube. If pollen was present in the corbicula, it was gently removed using tweezers and a needle under the microscope and transferred to a prepared, well-labeled PCR plate. The storage vial was also checked for any corbicular pollen adhering to its sides (i.e., compact, moist pollen which could be clearly associated with the corbicular pollen), which was added to the leg samples. If no pollen was detected on the legs or in the vials, this step was omitted. We first extracted the corbicular pollen before extracting the body pollen.

**Table 1:** Number of samples per species, transportation structure, landscape type and Swiss canton.

		Basel		Bern		Zurich		Total
		Urban	Rural	Urban	Rural	Urban	Rural	
*B. lapidarius*	Leg	21	41	10	11	43	26	152
	Body	101	116	46	20	102	104	489
*B. pascuorum*	Leg	50	41	30	22	54	41	238
	Body	117	107	89	49	108	107	577

Body pollen. We extracted body pollen on all 1066 bumblebee individuals (Table 1, Fig. 2). To collect the pollen, 500 μl of Milli-Q water was added to the bee in the Eppendorf tube, which was then briefly centrifuged and placed in an ultrasonic bath for 4 minutes. Following this, the Eppendorf tubes containing the bees were centrifuged at 10,000 RCF for 5 minutes. The bee was then removed and stored again at -20 °C. The remaining liquid and pollen mixture was centrifuged once more at 10,000 RCF for 1 minute to form a pellet, with the excess liquid discarded. The remaining liquid was then mixed with the pollen pellet and transferred to the PCR plate. The PCR plates were sealed with air-permeable tape and stored at -80 °C for at least 1 hour before being lyophilized overnight. The samples were then ready for metabarcoding and chemical analysis.

### DNA metabarcoding & bioinformatics

DNA metabarcoding (isolation, amplification, and sequencing) was performed by AllGenetics laboratories (AllGenetics & Biology SL; A Coruña, Spain). DNA was isolated from samples using the Quick-DNA Microprep Plus Kit (Zymo Research), strictly following the manufacturer’s instructions. DNA was resuspended in a final volume of 15 μL. A DNA extraction blank (Bex1-16) in each round of the DNA isolation procedure was included, to be treated as regular samples in the next step of the library construction process to check for cross-contamination. The isolated DNA was quantified by fluorimetry with Qubit, using the High-Sensitivity dsDNA Assay (Thermo Fisher Scientific). There was quantifiable DNA from most samples, except from 93 samples. These 93 samples were too low for Qubit quantification detection with the High-Sensitivity dsDNA Assay kit, meaning that the DNA values were below 0.1 ng/μL. Therefore, library construction may be compromised.

For library preparation, a fragment of the ITS2 genomic region (of around 300 bp) was amplified using the primers ITS_S2F^55^ and ITS4R^56^, as proposed and previously applied to metabarcoding^57^. Illumina sequencing primers were attached to these primers at their 5’ ends. Then, PCRs were carried out in a final volume of 12.5 μL, containing 1.25 μL of template DNA, 0.5 μM of the primers, 6.25 μL of Supreme NZYTaq 2x Green Master Mix (NZYTech), and ultrapure water up to 12.5 μL. In the next step, the reaction mixture was incubated as follows: an initial denaturation step at 95 °C for 5 min, followed by 35 cycles of 95 °C for 30 s, 51 °C for 45 s, 72 °C for 30 s, and a final extension step at 72 °C for 10 min. The oligonucleotide indices which are required for multiplexing different libraries in the same sequencing pool were attached in a second PCR round with identical conditions but only 5 cycles and 60 °C as the annealing temperature. A negative control that contained no DNA (BPCR) was included in every PCR round to check for contamination during library preparation. The libraries were then run on 2% agarose gels stained with GreenSafe (NZYTech), and imaged under UV light to verify the library size.

The libraries were purified using the Mag-Bind RXNPure Plus magnetic beads (Omega Biotek), following the instructions provided by the manufacturer. Then, the libraries were pooled in equimolar amounts according to the quantification data provided by the Qubit dsDNA HS Assay (Thermo Fisher Scientific). This pool also contained a testimonial amount of the corresponding extraction blanks (Bex) and the PCR blanks (BPCR). The pool was sequenced in a fraction of an Illumina NovaSeq PE250 flowcell (Illumina), aiming for a total output of 30 gigabases.

For bioinformatics, we followed the pipeline outlined at https://github.com/chiras/metabarcoding_pipeline^58^. This pipeline was implemented using VSEARCH v2.14.2^59^ for merging, quality truncation, and filtering (maxEE < 1; sequence length between 150 bp and 300 bp). Cleaned reads were then denoised into amplicon sequence variants (ASVs) and filtered for chimeras using VSEARCH. The ASVs were initially mapped using global alignments with VSEARCH against a floral ITS2 reference database specific to the study region, with an identity threshold of 97%. This database was created using BCdatabaser^60^ and included a list of potential plants found in the study area. For reads that remained unclassified, we used SINTAX^61^ to assign the deepest possible taxonomic levels using a global reference database^62^.

In DNA metabarcoding studies it has been observed that a low percentage of the reads of a library can be assigned to another library. This phenomenon, referred to as mistagging, tag jumping, index hopping, index jumping, etc. is the result of the misassignment of the indices during library preparation, sequencing, and/or demultiplexing steps^63–66^. ASVs were aggregated to species or genus level, depending on the deepest classification. In order to correct for this phenomenon, species occurring at a frequency below 0.01% in each sample were removed.

### Nutritional analyses

We focused on two critical macronutrients for bumblebee health and fitness: amino acids and fatty acids^67^. In order to have a sufficient pollen mass to perform the nutritional analyses, we pooled pollen (from the leg-baskets) from different bumblebee individuals collected by Eggenberger *et al*.^54^ within study sites and for each species separately. Overall, this resulted into 92 samples for fatty acid analyses and 85 for the amino acid analyses (Fig. 2).

#### Solvents and reagents needed for nutrient analyses

For the amino acid analysis:

- Deionized water
- Hydrochloric acid
- Sulfosalicylic acid
- Lithium buffer
- Amino acid calibration standard

For the fatty acid analysis:

- Nonadecanoic acid
- Chloroform
- Methanol
- Trimethylsulfohydroxide
- Fatty acid methyl ester standard

#### Amino acid analysis

We conducted amino acid analysis using ion exchange chromatography (IEC) with a Biochrom 20 plus amino acid analyzer, following the protocol by Kriesell *et al*.^68^. To analyze protein-bound amino acids (AA) in pollen, 5–10 mg of the collected pollen was first extracted in an ultrasonic bath with 100 µl of deionized water for 30 minutes. The extract was then refrigerated for 60 minutes, followed by centrifugation and membrane filtration for 10 minutes. The remaining residue, which was reserved for protein-bound amino acid analysis, was treated with 200 µl of 6 N HCl, boiled at 100 °C for 4 hours, cooled to room temperature, and centrifuged for 10 minutes. The supernatant was then subjected to evaporation at 100 °C to remove water and HCl, followed by re-dissolution and boiling in 200 µl of fresh water until dry. This process was repeated twice to ensure complete removal of the acid, but during the last time the sample was then centrifuged again before water was evaporated.

Next, 100 µl of the supernatant was mixed with 20 µl of 12.5% sulfosalicylic acid, frozen overnight, and extracted in the refrigerator for 30 minutes the following day. The mixture was briefly stirred, centrifuged for 10 minutes, and 100 µl of the supernatant was combined with 100 µl of sample rarefaction buffer (lithium buffer). This solution was then membrane-filtered by centrifugation for 5 minutes, and 20 µl of the filtrate was diluted in 80 µl of sample rarefaction buffer for IEC analysis. To quantify amino acids, we used the area of the peaks, calibrated via an external standard (physiological calibration standard from Laborservice Onken GmbH, Gründau, Germany) containing all proteinogenic amino acids except glutamine and asparagine, which were manually added before running the standards and samples (standard concentration was 200 µM per amino acid). The column used was a PEEK column packed with cation exchange resin. Amino acids were derivatized post-column via ninhdydrin and recorded via UV detection. Note that tryptophan could not be analyzed because it is destroyed by HCl.

#### Fatty acid analysis

Fatty acid (FA) analysis followed the protocol by Villagómez *et al*.^69^. For each pollen sample, 0.5 mg was homogenized with 7 µl of a 200 ng/µl solution of nonadecanoic acid in chloroform (used as an internal standard) in 0.1 ml of a 2:1 mixture of chloroform and methanol (both from Sigma-Aldrich, Taufkirchen, Germany). An additional 0.4 ml of the chloroform-methanol mixture was then added for further homogenization, and the mixture was transferred to a new vial with another 2.5 ml of the chloroform-methanol mixture, resulting in a total volume of 3 ml. The samples were shaken at 250 rpm for 24 hours and then evaporated to dryness. Subsequently, 10 µl of trimethylsulfohydroxide (TMSH) in 150 µl of dichloromethane (both from Sigma-Aldrich) was added, and the samples were analyzed using a gas chromatograph (GC, Agilent Series 8890) coupled to both a mass spectrometer (MS, Agilent 5977 C) and a flame-ionization detector (FID). Helium was used as the carrier gas (1 mL/min), and 1 µl of the sample was injected in splitless mode at 320 °C onto a fused silica column (Agilent Technologies DB5, 30 m length, 0.25 mm diameter). The oven temperature was initially set at 60 °C, increased to 150 °C at a rate of 15 °C/min, held for 10 minutes, then increased to 320 °C at 10 °C/min, and held for another 10 minutes.

Fatty acids were identified by comparing the mass spectra and retention times of peaks in the resulting chromatograms (MS) to standards (e.g., FAME C8-C24 and single fatty acid standards, Sigma-Aldrich), while the FID chromatograms were used to quantify fatty acids using the internal standard. In this method, di- and triglycerides are broken down into fatty acid methyl esters, so the FA content in this study refers to the total content of both free and glyceride-bound FAs. We also calculated the content and ratios of specific types of FAs relevant to bee nutrition, survival, and health^70^.

##### Metrics on nutrient intake

The total protein content was calculated as the sum of all amino acids, and the amino acid (AA) content in this study refers to the total protein-bound AA content. Additionally, we calculated the total content of essential AAs, which animals cannot synthesize and must obtain from their diet, as well as non-essential AAs, which animals can synthesize. The ratio between essential and non-essential AAs was also calculated. We calculated the content of saturated, unsaturated, omega-3, omega-6, and omega-9 FAs, as well as the ratios of saturated to unsaturated FAs and omega-3 to omega-6 FAs. These FA metrics are significant indicators of nutrition and health.

##### Metrics on diet breadth

We utilized taxonomic, functional, and phylogenetic metrics to assess bumblebee diet breadth at individual level (Table 2). Plant species richness served as the metric for plant taxonomic diversity^71^. For functional diversity, we calculated multidimensional metrics, focusing on three key dimensions: functional richness, functional evenness, and functional divergence, using the “FD” package (version 1.0–12.1) by Laliberté & Legendre^72^. We extracted plant traits (i.e., flowering duration^18^, flowering start^18^, growth form^18^, structural blossom class^18^, sugar concentration^51,52^, symmetry^18^ and plant height^18^) from multiple published, open-source datasets^18,51,52^. To compute functional diversity indices, we included flowering duration, structural blossom class, and pollen sugar concentration, as other traits present in the datasets (i.e., symmetry, flowering start, and plant height) had high correlations ( > 0.7). Moreover, besides the correlations, we limited the number of traits to three because the number of plant species in the pollen needed to exceed the number of traits, and most bumblebee individuals carried fewer than four plant species. Consequently, growth form was excluded to avoid filtering out too many bumblebee individuals.

**Table 2:** Number of samples for the functional and phylogenetic metrics of the diet breadth.

		Basel		Bern		Zurich		Total
		Urban	Rural	Urban	Rural	Urban	Rural	
*B. lapidarius*	Functional	10	15	5	1	25	13	69
	Phylogenetic	18	26	8	6	35	20	113
B. pascuorum	Functional	23	6	12	2	29	13	85
	Phylogenetic	38	19	19	8	41	26	151

For phylogenetic metrics, we calculated multidimensional indices, specifically phylogenetic variability, phylogenetic richness, phylogenetic evenness, and phylogenetic clustering, using the “picante” package (version 1.8.2) by Kembel *et al*.^73^, with the phylogeny from Jin and Qian^71^. Bumblebee individuals with fewer than four plant species in their collected pollen were excluded from the functional diversity analysis, as the convex hull could not be computed. For phylogenetic diversity metrics, individuals with fewer than three species were removed. In total, this resulted in 154 bumblebees being included for functional diversity analysis and 264 individuals for phylogenetic diversity analysis (Fig. 2).

## Data Records

DNA reads from the metabarcoding analyses were uploaded and archived in the National Centre of Biotechnology Information (NCBI)^74^.

The remaining data records are uploaded and available from the repository ENVIDAT, the Swiss data portal for environmental monitoring and research data^75^. It includes the following processed datasets: 1) pollen community composition; 2) study sites; 3) nutrient intake 1: amino acids; 4) nutrient intake 2: fatty acids; 5) diet breadth 1: species richness; 6) diet breadth 2: functional diversity; 7) diet breadth 3: phylogenetic diversity; 8) plant traits. The dataset on pollen community composition (dataset1_metabarcoding_pollen.csv)contains the plant species and their relative abundance in both the body and leg-baskets for each bumblebee individual (Table 3). The dataset on the study sites (dataset2_sites.csv) provides the codes, geographic coordinates of the 800-m centroid, as well as the plant composition and the proportion of impervious, agricultural, water and other habitats within the buffer (Table 4). The datasets on nutrient intake (dataset3_aminoacids.csv and dataset4_fattyacids.csv and) (Tables 5, 6) contain the contents of individual AAs and FAs as well as the contents and ratios of the studied groups of AAs and FAs (see Tables 5, 6). The datasets on diet breadth (dataset5_species_richness.csv, dataset6_functional_diversity.csv and dataset7_phylogenetic_diversity.csv) (Tables 7–9) contain the taxonomic diversity (i.e., species richness, Table 7), functional diversity (i.e., functional richness, evenness and dispersion, weighted and unweighted, Table 8) and phylogenetic diversity (i.e., phylogenetic variability, richness, evenness and clustering, Table 9) metrics. Each row represents a bumblebee individual. The dataset on plant traits (dataset8_planttraits.csv) results from combining the published information in previous datasets^18,51,52^ (Table 10), presenting different traits depicting origin status, flowering (e.g., floral accessibility, rewards, phenology, Table 10) and overall plant descriptors (e.g., plant height, growth form, Table 10).

**Table 3:** The data structure of the metabarcoding dataset.

Variable name	Description	Variable type
SampleID	ID, composed of bumblebeeID, location, landscape and replicate	Character
Abundance	Relative abundance of each plant species	Numeric
BumblebeeID	ID of each bumblebee indiviudual, nested to the location, landscape and replicate sampling location	Numeric
Location	The three swiss cantons included in the sampling	Factor, levels: Bern = BE; Basel = BS; Zurich = ZH
Landscape	The two typea of human-dominated ecosystems, nested to the Swiss canton	Factor, levels: Urban = U; Rural = R
Replicate	Sampling place, nested to the location and the landscape	Character, letters A-E
LocationLandscape	Combination of the location and landscape	Factor
Bumblebee_species	The two bumblebee species studied	Factor, levels: Bombus pascuorum = B.pasquorum; Bombus lapidarius = B. lapidarius
Transportation structure	The two bumblebee pollen transportation from which pollen was extracted separately	Factor, levels: B = Body; L = leg-baskets (i.e. corbicula)
Kingdom	Constant, Viridiplantae	Factor
Phylum	Plant phylum	Factor
Order	Plant Order	Factor, multiple levels
Family	Plant Family	Factor, multiple levels
Genus	Plant Genus	Factor, multiple levels
Species	Plant scientific name	Factor, multiple levels
Binomial_abundance	Always 1, indicating the species had enough abundance	Numeric

**Table 4:** The data structure for the site information dataset.

Variable name	Description	Variable type
BumblebeeID	ID of each bumblebee indiviudual, nested to the location, landscape and replicate sampling location	Numeric
Location	The three swiss cantons included in the sampling	Factor, levels: Bern = BE; Basel = BS; Zurich = ZH
Landscape	The two typea of human-dominated ecosystems, nested to the Swiss canton	Factor, levels: Urban = U; Rural = R
Replicate	Sampling place, nested to the location and the landscape	Character, letters A-E
Site_ID	Combination of location, landscape and replicate	Factor
X	X coordinate, WGS84	Numeric
Y	Y coordinate WGS84	Numeric
Plant S in landscape	Plant species richness within the 800 m calculated using both information from the Global Biodiversity Information. Facility (GBIF) and InfoFlora (www.infoflora.ch)	Numeric
% Buildings	The proportion of buildings and other impervious surfaces within 800 m radius	Numeric
% Agricultural	The proportion of agricultural land and other impervious surfaces within 800 m radius	Numeric
% Water	The proportion of water and other impervious surfaces within 800 m radius	Numeric
% Meadows & grasslands	The proportion of meadows and grasslands and other impervious surfaces within 800 m radius	Numeric

**Table 5:** The data structure for the amino acid dataset.

Variable name	Description	Variable type
Bumblebee_species	The two bumblebee species studied	
Location	The three swiss cantons included in the sampling	Factor, levels: Bern = BE; Basel = BS; Zurich = ZH
Landscape	The two typea of human-dominated ecosystems, nested to the Swiss canton	Factor, levels: Urban = U; Rural = R
LocationLandscape	Combination of the location and landscape	Factor
Replica	Sampling place, nested to the location and the landscape	Character, letters A-E
SampleId_chemical	Sampling ID for chemical analyses	Character
Asp, …, Arg	Content of individual amino acids (mg/g)	Numeric
sum	Total sum of amino acid content (mg/g)	Numeric
sumessential	Total sum essential amino acids (mg/g)	Numeric
sumnon-essential	Total sum non-essential amino acids (mg/g)	Numeric
proportionnonessentialessential	Proportion between essential and non-essential amino acids	Numeric

Amino acid names are written in the abbreviated form. Essential amino acids for bees: arginine, histidine, isoleucine, leucine, lysine, methionine, phenylalanine, threonine, tryptophan and valine (De Groot, 1952).

**Table 6:** The data structure of the fatty acid dataset.

Variable name	Description	Variable type
Bumblebee_species	The two bumblebee species studied	
Location	The three swiss cantons included in the sampling	Factor, levels: Bern = BE; Basel = BS; Zurich = ZH
Landscape	The two types of human-dominated ecosystems, nested to the Swiss canton	Factor, levels: Urban = U; Rural = R
Replica	Sampling place, nested to the location and the landscape	Character, letters A-E
SampleID_chemistry	Sampling ID for chemical analyses, resulting from merging pollen from different bumblebee individuals within the same location, landscape and replica	Character
Caprylic, …, Lacceroic	Content of the individual fatty acids (mg/g)	Numeric
Sum	Total sum of fatty acid content (mg/g)	Numeric
unsaturatedsum	Total sum unsaturated fatty acids (mg/g)	Numeric
saturatedsum	Total sum saturated fatty acids (mg/g)	Numeric
omega3sum	Total sum Omega 3 fatty acids (mg/g)	Numeric
omega6sum	Total sum Omega 6 fatty acids (mg/g)	Numeric
omega9sum	Total sum Omega 9 fatty acids (mg/g)	Numeric
omega6to3ratio	Ratio Omega 6 to Omega3 fatty acids	Numeric
saturatedtounsaturatedratio	Ratio between saturated and unsaturated fatty acids	Numeric

**Table 7:** The data structure of the plant species richness (diet breadth - taxonomic).

Variable name	Description	Variable type
SampleID	ID, composed of	Character
Location	The three swiss cantons included in the sampling	Factor, levels: Bern = BE; Basel = BS; Zurich = ZH
Landscape	The two typea of human-dominated ecosystems, nested to the Swiss canton	Factor, levels: Urban = U; Rural = R
Replicate	Sampling place, nested to the location and the landscape	Character, letters A-E
LocationLandscape	Combination of the location and landscape	Factor
Bumblebee_species	The two bumblebee species studied	Factor, levels: Bombus pascuorum = B.pasquorum; Bombus lapidarius = B. lapidarius
Plant species richness	The number of plant species found in the pollen of the leg-baskets	Numeric

**Table 8:** The data structure of the functional diversity metrics (diet breadth functional).

Variable name	Description	Variable type
SampleID	ID, composed of	Character
Location	The three swiss cantons included in the sampling	Factor, levels: Bern = BE; Basel = BS; Zurich = ZH
Landscape	The two typea of human-dominated ecosystems, nested to the Swiss canton	Factor, levels: Urban = U; Rural = R
Replicate	Sampling place, nested to the location and the landscape	Character, letters A-E
LocationLandscape	Combination of the location and landscape	Factor
Bumblebee_species	The two bumblebee species studied	Factor, levels: Bombus pascuorum = B.pasquorum; Bombus lapidarius = B. lapidarius
FRic.w	Weighted functional richness	Numeric
FRic	Functional richness	Numeric
FEve.w	Weighted functional evenness	Numeric
FEve	Functional evenness	Numeric
FDis.w	Weighted functional dispersion	Numeric
FDis	Functional dispersion	Numeric

**Table 9:** The data structure of the phylogenetic diversity metrics (diet breadth phylogenetic).

Variable name	Description	Variable type
SampleID	ID, composed of	Character
Location	The three swiss cantons included in the sampling	Factor, levels: Bern = BE; Basel = BS; Zurich = ZH
Landscape	The two types of human-dominated ecosystems, nested to the Swiss canton	Factor, levels: Urban = U; Rural = R
Replicate	Sampling place, nested to the location and the landscape	Character, letters A-E
LocationLandscape	Combination of the location and landscape	Factor
Bumblebee_species	The two bumblebee species studied	Factor, levels: *Bombus pascuorum* = *B.pasquorum*; *Bombus lapidarius* = *B. lapidarius*
PVar	Phylogenetic variance	Numeric
PRic	Phylogenetic richness	Numeric
PEve	Phylogenetic evenness	Numeric
PClu	Phylogenetic clustering	Numeric

**Table 10:** The data structure of the plant traits dataset.

Variable name	Description	Variable type
Kingdom	Constant, Viridiplantae	Factor
Phylum	Plant phylum	Factor
Order	Plant order	Factor, multiple levels
Family	Plant family	Factor, multiple levels
Genus	Plant genus	Factor, multiple levels
Species	Plant species	Factor, multiple levels
Plant.species	Plant scientific name	
native_exotic	Origin status	Factor. Levels: N = native, E = non-native
pollination_mode		
Flowering_months_duration	Duration flowering in months	Numeric
start_flowering	Starting month of the flowering	Numeric
growth_form_category	Growth form	Factor. Levels: herbs, climbers, shrubs, trees
plant_height_m	Measure of the height of a plant species in meters.	Numeric
inflorescence	Determines whether the blossom is a single flower or an inflorescence.	Logic, single flowers (0), inflorescences (1)
structural_blossom_class	Describing the shape of the blossom of the plant species.	Factor. Levels: dish-bowl, stalk-disk, bell trumpet, brush, gullet, flag, tube
symmetry	Describes the number of axes of reflection of a flower of a plant species. The value was derived from the structural blossom class	Factor. Levels: no symmetry, zygomorph, actinomorph
nectar	Describes whether the plant provides nectar resources.	Logic, abscent (0), present (1)
pollen	Describes whether the plant provides pollen resources.	Logic, abscent (0), present (1)
oil	Describes whether the plant provides oil resources.	Logic, abscent (0), present (1)
sugar.concentration	Describes the concentration of sugar in nectar, in mg.	Numeric

## Technical Validation

A negative control that contained no DNA (BPCR) was included in every PCR round to check for contamination during library preparation. The libraries were run on 2% agarose gels stained with GreenSafe (NZYTech), and imaged under UV light to verify the library size. Raw sequencing data quality was assessed first using FastQC (https://www.bioinformatics.babraham.ac.uk/projects/fastqc) to obtain information about the sequencing run separately for each sample. The results seemed appropriate according to previous experience with published protocols^57^ and other applied studies^76,77^ for the ITS2 marker. In data processing, quality was assessed in several steps and accordingly used for filtering out erroneous data (see Method section). As aforementioned, in order to correct for mistagging (as a result of the misassignment of the indices during library preparation, sequencing, and/or demultiplexing steps), species occurring at a frequency below 0.01% in each sample were removed.

## Usage Notes

**Fig. 3:**
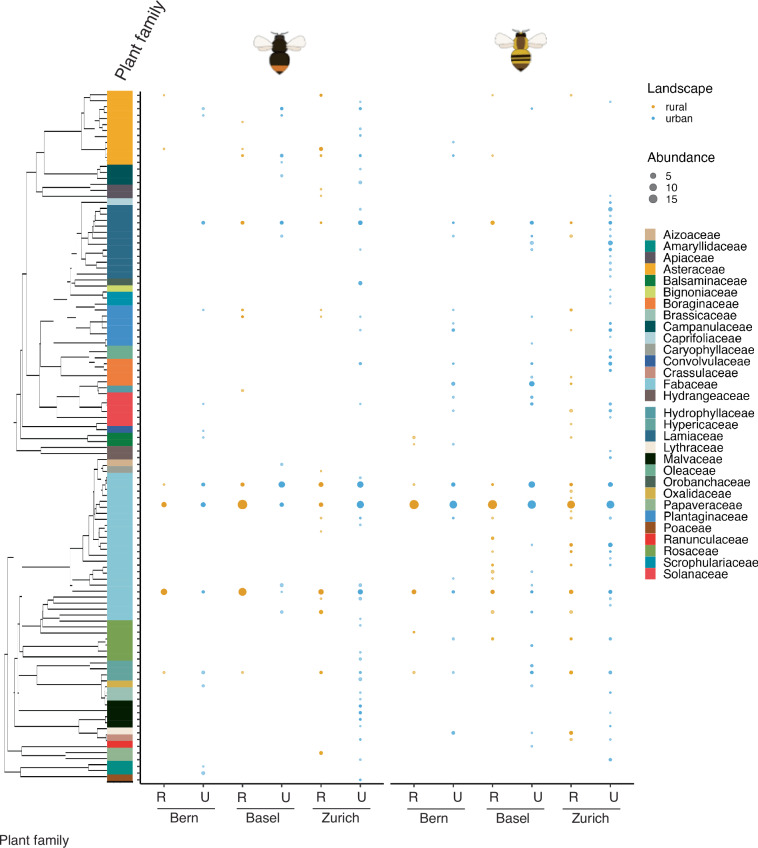
Plant composition in the leg-basket pollen from urban and rural bumblebee individuals. Cumulative abundance of the different plant species present in the pollen according to the location (Swiss canton), landscape (urban or rural) and bumblebee species (*Bombus lapidarius*, left; *B. pascuorum*, right). Size of the dots indicates the cumulative sum of relative abundances per bumblebee individual. Plant species are grouped in families. R = Rural, U = Urban.

**Fig. 4:**
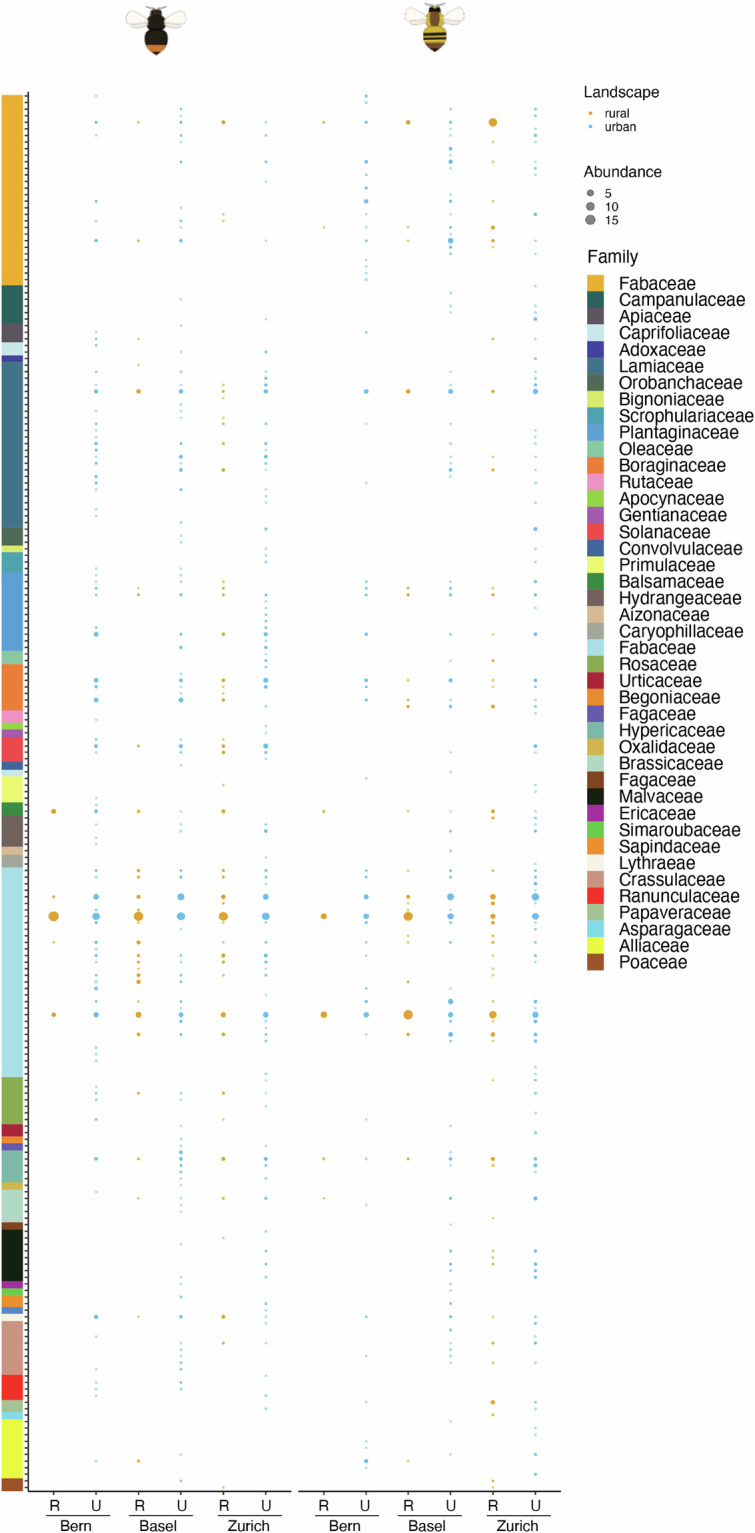
Plant composition in the body pollen from urban and rural bumblebee individuals. Cumulative abundance of the different plant species present in the pollen according to the location (Swiss canton), landscape (urban or rural) and bumblebee species (*Bombus lapidarius*, left; *B. pascuorum*, right). Size of the dots indicates the cumulative sum of relative abundances per bumblebee individual. Plant species are grouped in families. R = Rural, U = Urban.

The datasets included in this publication can already be used by researchers as provided. The plant composition data obtained from pollen metabarcoding can be compared to other data or for comparison in future study by two ways: 1) Data inferred using similar metabarcoding criteria (e.g., targeted regions, sequencing technology) can directly reuse the raw sequencing data for a combined processing in a meta-analysis. 2) The processed data can be reused and compared to such originating from other methods, such as observation networks. Moreover, the plant composition data (Figs. 3, 4) can be used to assemble plant-bumblebee individual interaction networks, which can later be used to study network properties and metrics. Metrics on diet breadth, particularly on functional and phylogenetic dimensions, can be recalculated as plant phylogenies get updated or via the inclusion of alternative traits. In that regard, as more plant trait databases become more accessible, specifically regarding floral attractiveness, phenology and nutritional content, new metrics can be calculated.

## Data Availability

Code to calculate the functional and phylogenetic diet breadth can be found in https://github.com/joancasanelles/Data_bumblebee_diet. Code for bioinformatic analyses is available at https://github.com/chiras/metabarcoding_pipeline.
